# Postpartum depression screening scale-7: A valid and reliable short version both for portugal and brasil

**DOI:** 10.1192/j.eurpsy.2021.1618

**Published:** 2021-08-13

**Authors:** A.T. Pereira, M. Barros, M. Aguiar, J. Azevedo, M. Marques, F. Carvalho, D. Pereira, A. Macedo

**Affiliations:** 1 Institute Of Psychological Medicine, Faculty Of Medicine, University of Coimbra, coimbra, Portugal; 2 Departamento De Ciências Naturais, Universidade do Sudoeste da Bahia - UESB, Vitória da Conquista, Bahia, Brazil; 3 Pos Graduação Em Psicologia Da Saúde, Universidade Federal da Bahia, Vitória da Conquista, Brazil; 4 Institute Of Psychological Medicine, Faculty Of Medicine, University of Coimbra, Coimbra, Portugal

**Keywords:** PDSS-7, Perinatal depression, Postpartum depression

## Abstract

**Introduction:**

Screening programs for perinatal depression are systematicly implemented in developed countries. To circumvent the most commonly pointed limitation by the primary healthcare professionals (the questionnaires length), we have developed shorter forms of the Beck and Gable Postpartum Depression Screening Scale-35. The shortest version consists of seven items, each one representing a dimension evaluated by the PDSS. This PDSS-7 demonstrated equal levels of reliability and validity as the 35-item PDSS with the advantage of being completed in as little as 1-2 minutes(Pereira et al. 2013).

**Objectives:**

To analyze the construct validity of the PDSS-7 using Confirmatory Factor Analysis, to use both in Portugal and in Brazil.

**Methods:**

The Portuguese sample was composed of 616 women (Mean age: 32.29±4.466; Mean gestation weeks=17.13±4.929). These participants were not the same who participated in the psychometric study that led to the selection of the seven items. The Brazilian sample was composed of 350 women (Mean age: 30.01±5.452; Mean gestation weeks=25.17±6.55). They all had uncomplicated pregnancies and completed the European/Brazilian Portuguese versions of PDSS-24 (Pereira et al. 2013/ Barros et al. 2021), which was composed of the same items and included the seven items that compose the PDSS-7.

**Results:**

The unidimensional model of PDSS-7 presented a good fit in both samples (Portuguese/Brazilian: χ^2^/d.f.=3.439/2.653; RMSEA=.066/.069, CFI=.974/.981, TLI=.947/.957, GFI=.939/.957; p<.001). The PDSS-7 Cronbach’s alphas were .82/.83 and all the items contribute to the internal consistency.

**Conclusions:**

The PDSS-7 is a valid and precise, economic, fast and easy screening instrument for perinatal depression, a major public health problem, both in Portugal and in Brazil.

